# TRPV3: Structure, Diseases and Modulators

**DOI:** 10.3390/molecules28020774

**Published:** 2023-01-12

**Authors:** Wuyue Su, Xue Qiao, Wumei Wang, Shengnan He, Ke Liang, Xuechuan Hong

**Affiliations:** State Key Laboratory of Virology, College of Science, Research Center for Ecology, Laboratory of Extreme Environmental Biological Resources and Adaptive Evolution, Medical College, Tibet University, Lhasa 850000, China

**Keywords:** TRPV3, structure, diseases, agonists, antagonists

## Abstract

Transient receptor potential vanillin 3 (TRPV3) is a member of the transient receptor potential (TRP) superfamily. As a Ca^2+^-permeable nonselective cation channel, TRPV3 can recognize thermal stimulation (31–39 °C), and it plays an important regulatory role in temperature perception, pain transduction, skin physiology, inflammation, cancer and other diseases. TRPV3 is not only activated by the changes in the temperature, but it also can be activated by a variety of chemical and physical stimuli. Selective TRPV3 agonists and antagonists with regulatory effects and the physiological functions for clinical application are highly demanded. In recent years, significant progress has been made in the study of TRPV3, but there is still a lack of modulators with a strong affinity and excellent selectivity. This paper reviews the functional characteristics of TRPV3 in terms of the structure, diseases and the research on TRPV3 modulators.

## 1. Introduction

**Figure 1:**
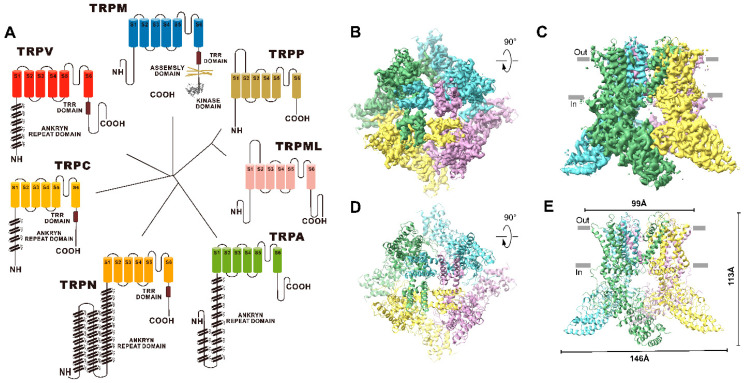
The structural diagram of the TRP channel subfamily, its main subunits (**A**) and top (**B**,**D**) and side (**C**,**E**) views of the 3D cryo-EM reconstruction of TRPV3 in the apo (closed) state [20,21]. Reprinted with permission from Ref. [20]. 2015, Springer Nature.

**TRP channels:** Transient receptor potential (TRP) channels are a class of nonselective cationic channel proteins that are widely distributed in the peripheral and central nervous systems. TRPs were first identified in the mutant Drosophila melanogaster with impaired visual transmission [1]. In 1975, Minke named TRP for the first time based on its electrophysiological phenotypes [2]. The TRP superfamily plays important roles in humans, Drosophila, mice and worms. The TRP superfamily proteins consist of several significant sequence elements and domains, and the amino-(*N*-) and carboxy-(C-) terminus structures are composed of a cytoplasmic ankyrin repeat domain (ARD), a connector domain, an S1–S4 domain, an S5–S6 pore domain, a tryptophan domain and a C-terminal domain (Figure 1) [3,4]. The TRP channels consist of seven subfamilies: TRPM subfamily (M for melastatin), TRPC subfamily (C for canonical), TRPA subfamily (A for ankyrin), TRPV subfamily (V for vanilloid), TRPP subfamily (P for polycystin), TRPML subfamily (ML for mucolipin) and TRPN subfamily (N for nonmechanoreceptor). Although the TRP superfamily is defined according to its structural characteristics, the amino acid sequence identity value of all of the TRP families is ~20%, which mainly covers the transmembrane structure. The TRP superfamily has been found to participate in sensory transduction pathways, including light perception, temperature perception, mechanical perception and chemical perception [5,6,7,8]. In particular, the TRP channel, on the basis of pain and temperature perception, has attracted a lot of attention recently [6,9,10]. In addition, the TRP superfamily is involved in many other physiological processes, including Ca^2+^ and Mg^2+^ homeostasis, stress regulation, lysosomal function, inflammation, cardiovascular regulation, salivary secretion and smooth muscle tone functions [11,12,13,14,15,16,17,18]. The TRP superfamily is also closely associated with human diseases because of its involvement in many physiological processes [19].

**Figure 2:**
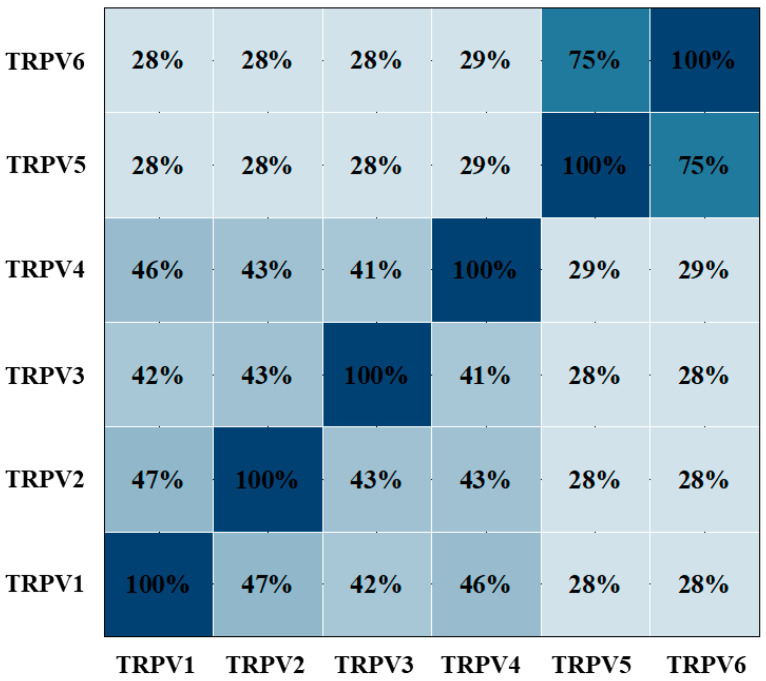
The similarity between TRPV1-6.

**TRPV subfamily:** The TRPV channel protein is a member of the TRP superfamily that was first identified in 1997 and systematically reported in 2001 [11,22]. The TRPV subfamily is named for the vanilloids (e.g., capsaicin) that activate the first member of this subfamily, TRPV1. The TRPV subfamily includes TRPV1, TRPV2, TRPV3, TRPV4, TRPV5 and TRPV6 ion channels with different functions [23,24,25,26]. TRPV3 ion channels have a low sequence identity value compared with other TRPV ion channels. The sequence identity values of the TRPV1, TRPV2, TRPV4 and TRPV5/6 channels is ~42%, ~43%, ~41% and ~28%, respectively [26]. In addition, TRPV2, TRPV3, TRPV4 and TRPV5/6 share ~47%, ~42%, ~46% and ~28% similarity with TRPV1, respectively (Figure 2) [26,27]. TRPV3 is a temperature-sensitive and Ca^2+^-permeable nonselective cation channel that forms a tetramer complex through six transmembrane domains [24]. Like other calcium channel proteins, TRPV3 channels exert physiological functions and influence the body by controlling the Ca^2+^ flow into the cells through the stimulation of extracellular signals. The opening of these channels may lead to the increase in the intracellular calcium concentration and the membrane depolarization of the receptor cells, thereby promoting the release of action potentials and the activation of intracellular signal transduction mechanism, thus regulating the physiological function of the cells. In 2018, Appu K. Singh et al. successfully revealed the cryo-electron microscopy structure and gating mechanism of mouse TRPV3 channels in open and closed states for the first time (Figure 1) [21]. TRPV3 is known to have a temperature activation threshold of 31–39 °C. The molecular mechanism of TRPV3 thermal activation is not fully understood. Grandl et al. screened 14,000 random mutant clones and found that five amino acid residues (N643, I644, N647, L657 and Y661) located in the transmembrane segment of S6 and the adjacent pore loop play an important role in the thermal activation of TRPV3 [28]. Sung Eun Kim et al. found that I652 and L655 also mediated the thermal response of TRPV3 near the above sites [29]. TRPV3 can also be activated or inhibited by certain endogenous ligands, natural products, and small-molecule synthetic chemicals. Changes in the extracellular cations, pH, membrane voltage and other physiological factors can regulate the function of TRPV3. TRPV3 is distributed throughout various tissues, including the intestinal epithelial cells, vascular endothelial cells, epidermal keratinocytes, oral epithelial cell, corneal epithelial cells, dorsal root ganglion and central nervous system neurons [30,31]. In contrast to other TRP channels in the same family, TRPV3 channels show unique sensitization rather than desensitization upon repeated stimulation [32]. TRPV3 channels play an important role in maintaining the normal physiological functions of the body, and they have been found to be involved in temperature and pain perception. In addition, TRPV3 channels also play an important role in the generation of skin and hair. TRPV3-null mice exhibit impaired skin function, including hair growth, skin barrier formation, wound healing, skin pain and itch production [24,26,33,34,35,36]. Finding and screening TRPV3 modulators with high activity and selectivity may provide a new way of treating related diseases.

## 2. Diseases Related to TRPV3

Since TRPV3 plays an indispensable role in the generation of pain, the research on TRPV3 in early studies mainly focused on pain sensation and thermal sensation. With further studies on physiological functions having been conducted, TRPV3 has been found to be associated with a variety of diseases (Table 1).

### 2.1. Skin Diseases

The TRPV3 channel is essential for skin barrier formation, keratinocyte maturation, wound healing and itching. The overexpression of TRPV3 can lead to hair loss, skin inflammation, severe pruritus and dermatitis [42]. Olmsted syndrome (OS), also known as dermal keratosis and perioral dermatosis, is a rare keratosis with autosomal dominant inheritance. Exon sequencing has revealed that an autosomal dominant heterozygous missense mutation encoding TRPV3 causes OS. It is characterized by bilateral palmoplantar keratosis (PPK) and peripheral keratosis plaques, and all of them cause severe pruritus [41,60]. Studies applying the patch-clamp analysis technique to TRPV3-expressing mutants have shown that these mutations lead to greater inward currents, resulting in elevated intracellular Ca^2+^ and induced increased apoptosis in the epidermal cells, suggesting that increased cell death may drive OS [61]. The clinical treatment of OS is usually administered with antidepressants, opioids, anticonvulsants and nonsteroidal anti-inflammatory drugs. The topical treatments include antidotes, retinoic acid and corticosteroids. The systemic treatments includes the use of norepinephrine or retinoic acid. Surgical treatments are performed on regular scabs using skin grafts [60]. It was reported that the EGFR inhibitor, Erlotinib, has been reported clinically for the treatment of OS [60]. However, these treatments are ineffective and prone to relapse. OS is a disease that is caused by the TRPV3 gene mutation, and TRPV3 gene knockout mice have demonstrated significant pruritus inhibitory behavior [41]. A large number of studies have shown that the topical application of TRPV3 inhibitors can effectively inhibit skin pruritus and pain, which provides a new idea for the treatment of intractable pruritus and pain diseases such as Olmsted syndrome and hereditary palmoplantar keratosis.

**Figure 3:**
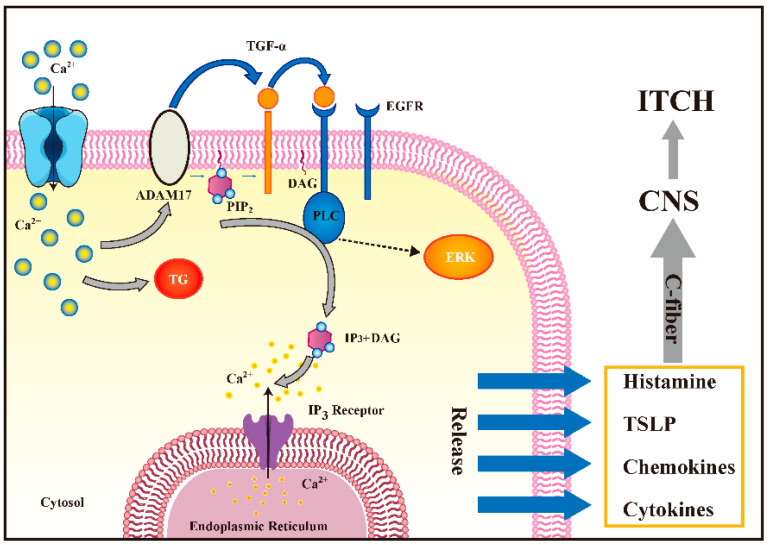
The role of TRPV3 in itch signal and cell proliferation. ERK, extracellular signal-regulated kinase; ADAM17, metalloprotease ADAM17; DAG, diacylglycerol; IP3, inositol trisphosphate; PIP2, phosphatidylinositol(4,5)bisphosphate; PLC, phospholipase C; CNS, central nervous system; TSLP, thymic stromal lymphopoietin [34].

In addition to hereditary Olmsted syndrome pruritus, TRPV3 is closely associated with the development and progression of various skin diseases since it is significantly increased in the epidermal cells of patients with acquired skin diseases such as rosacea, psoriasis and atopic dermatitis. Studies on the skin tissues of burn patients showed that the expression of TRPV3 was significantly upregulated in the epidermis of patients with post-burn pruritus [48]. The activation of TRPV3 ion channels in keratinocytes resulted in the release of histamine, TSLP, chemokines and cytokines, which further stimulated the sensory input of adjacent skins by acting as intercellular messengers, thereby inducing skin pain and itching (Figure 3). The inhibition of the TRPV3 channels is a new idea for the treatment of skin diseases, and many scientists have begun to focus on the discovery of TRPV3 inhibitors [62,63,64,65].

### 2.2. Cancers

It has been reported that the high expression of TRPV3 will cause pain in pancreatic cancer, bone cancer and breast cancer [21]. Patients often experience pain before breast cancer surgery, and there is evidence that this pain is due to the high expression of TRPV3 [56,66,67,68]. Birgit Hoeft et al. analyzed 392 single nucleotide polymorphisms of 43 genes associated with fatty acid metabolism and the risk of colorectal cancer in a study involving 1225 cancer patients and 2032 noncancer controls. TRPV3 was also shown to be associated with a higher risk of colorectal cancer [69]. Mehmet Emin Kalender et al. analyzed the tissues of 37 females and 56 males with colorectal cancer and found that the mRNA expression level of the TRPV3 gene was much lower than it was in the healthy tissues (*p* < 0.05) [52]. Xiaolei Li et al. also found that TRPV3 was highly expressed in 65 out of 96 cases of lung cancer (67.7%). In general, TRPV3 expression varies across different cancers. Statistically, TRPV3 expression is decreased in colorectal cancer, and it is increased in pancreatic cancer, bone cancer, breast cancer, lung cancer and oral squamous cell carcinoma [21,50,52,70]. Studies have shown that TRPV3 may increase the Ca^2+^ concentration in cancer cells, activate calmodulin kinase, and then, it may affect the cell cycle and promote cell proliferation [71]. Importantly, the inhibition of TRPV3 slowed the proliferation of lung cancer cells [50]. In conclusion, inhibiting the expression of TRPV 3 provides a new strategy for the treatment of cancer.

### 2.3. Cardiac Diseases

Studies have identified TRPV3 as a key regulator of cardiac diseases. Hongli Sun et al. revealed the role of TRPV3 in the course of cardiac hypertrophy cases. TRPV3 was elevated in pathological cardiac hypertrophy, and TRPV3 expression was also increased in Ang II-induced cardiomyocyte hypertrophy in vitro. Meanwhile, the cellular experiments have shown that the non-selective TRPV3 inhibitor, carvacrol, can significantly aggravate cardiomyocyte hypertrophy, while the non-selective TRPV3 antagonist, ruthenium red, can slow down cardiomyocyte hypertrophy. Further studies have showed that TRPV3 activation aggravated pathological cardiac hypertrophy through calcineurin/NFATc3 signaling [53]. In addition, the inhibition of TRPV3 can reduce the amount of intracellular calcium ions and further decrease cardiac autophagy activity, thereby reducing cardiomyocyte hypertrophy [72]. A series of results have suggested that TRPV3 may be a potential therapeutic target for myocardial hypertrophy. Hongli Sun et al. further found that TRPV3 activation promoted the proliferation of cardiac fibroblasts through the β-CDK1/CDK2/Cyclin E pathway, thereby aggravating cardiac fibrosis [54]. Hypoxia-induced apoptosis and inflammation are important causes of cardiovascular diseases such as myocardial infarction (MI), and TRPV3 siRNA can protect the cardiomyocytes from hypoxia-induced apoptosis and inflammation [55]. The current results fully demonstrated that TRPV3 may play a certain role in the occurrence and development of cardiovascular diseases. Further elucidation of the related mechanisms will provide new intervention targets for the prevention and treatment of cardiovascular diseases. There are no TRPV3 antagonists in the current clinical trials, but there is evidence that TRPV3 may be a new direction for the treatment of cardiac diseases.

### 2.4. Others

TRPV3 is also expressed in human and mouse corneal epithelial cells, and it plays an important role [31]. Ocular lesions including corneal dystrophy, corneal epithelial dysplasia and lacrimal gland inflammation are common in OS patients with TRPV3 mutations [73]. It has been reported that human corneal epithelial cells (HCEC) can activate heat-sensitive TRPV3, and TRPV3 activation can protect the cornea against harmful increases in the ambient temperature [74]. Wound healing assays have showed that activation of TRPV3 in corneal epithelial cells accelerated the proliferation of corneal epithelial cells [31]. Interestingly, the activation of TRPV3 in oral epithelial cells by heat induction can accelerate the proliferation of oral epithelial cells and wound healing [75]. Exploring its mechanism showed that TRPV3 and EGFR formed a signaling complex, and TRPV3 activation could enhance the activity of EGFR signaling, and then, regulate epithelial cell proliferation [76]. A positive feedback loop was formed between TRPV3 and TGF-α/EGFR, possibly leading to terminal differentiation of the basal keratinocytes (Figure 3). This may also explain why the overexpression of TRPV3 has been detected in many cancer cells.

In addition, TRPV3 is closely related to pain. The overexpression of TRPV3 has been found in skin pain, breast pain, cancer pain and other pain tissues [56,57,58]. The relevant evidence has shown that the TRPV3 channels in the epidermis are involved in sensory conduction, but the TRPV3 channels in the skin keratinocytes do not form synapses with the sensory nerve terminals. It was speculated that small molecules may form a “bridge” between the epidermal cells and sensory nerve terminals to complete signal transmission. When the body is injured by the outside world, the injured tissue site will promote the release of prostaglandin E2 (PGE2), ATP, NO, interleukin 1α (Il-1α), TGF-β and other substances, which sensitize the sensory nerve endings of the skin [36,58]. Complete signal transmission results in specific physiological effects.

The TRPV3 agonist, carvacrol, inhibits hair growth, and another TRPV3 inhibitor, forsythoside B, significantly reverses it [59]. TRPV3 signaling plays an important role in the control of human hair growth, which may be a promising target for the development of drugs for hair growth disorders [77].

## 3. Small-Molecule TRPV3 Modulators

In recent years, a number of small-molecule modulators including natural and synthetic compounds were identified to show specific effects on various functions through acting at the TRPV3 channels in academic and pharmaceutical industry laboratories.

### 3.1. TRPV3 Agonists

#### 3.1.1. Natural Compounds

Camphor (Table 2, entry 3) is one of the early natural compounds that was identified to activate TRPV1, TRPV3, TRPM8 and TRPA1 channels [78,79]. The activation of TRPV1 and TRPV3 channels could enhance thermal sensitivity, and the activation of TRPM8 could induce cold sensitivity [80]. In addition, the TRPV3 channels in the brain may also play a role in mood regulation. Incensole acetate (IA) (Table 2, entry 1), isolated from *Boswellia* resin, was an effective TRPV3 agonist to induce anti-anxiety and anti-depressive behavior effects in wild-type (WT) mice with an EC_50_ of 16 μM [81]. Additional research has suggested that incensol may be a good candidate for neuroprotection because of its multipotent active mechanisms, including modulating TRPV3 and the pro-inflammatory transcription factor STAT3 [82]. Serratol (Table 2, entry 2), an analog of IA isolated from Indian frankincense, showed more TRPV3 inhibitory activity, with an IC_50_ of 0.15 ± 0.01 μM [82]. Haoxing Xu et al., using Ca^2+^ imaging and patch-clamp recordings, demonstrated that carvacrol (Table 2 entry 6), thymol (Table 2, entry 10) and eugenol (Table 2, entry 7) can activate the TRPV3 channels that are allogenically expressed in HEK293 cells and the endogenous TRPV3 channels in epithelial cells and keratinocytes [63]. In addition, carvacrol can activate TRPA1, and Eugenol can activate TRPV1 [63,83]. A recent study reported that carvonol interacts with L508, a key residue in the S2–S3 linker, leading to conformational change in or the conformational rearrangement and opening of the TRPV3 channels [84]. Citral, a bioactive component of lemongrass, can activate the TRPV1, TRPV3, TRPM8 and TRPA1 channels. It has a long-term inhibitory effect on TRPV1-3 and TRPM8 channels, and it can temporarily block the TRPV4 and TRPA1 channels. The broad spectrum and long-term sensory inhibitory activity of citral may be more effective than that of capsaicin in the treatment of abnormal pain, pruritus or other types of pain involving the superficial sensory nerves and skin [85]. AK Vogt-Eisele et al. found more than 30 compounds with excitatory effects on TRPV3 by screening the natural chemical library of monoterpenoids. Six compounds with a ring-located hydroxyl group have better TRPV3 activity than camphor does (Table 2, entries 3–4, 6, 9–12) [86]. A structure–activity relationship study discovered that the secondary hydroxyl group was the structural requirement for the effective activation of TRPV3. The oxidation of the secondary hydroxyl to the carbonyl group will significantly reduce the activity of the substance. In addition, Hanns Hatt et al. found that monoterpenoids can strongly activate the TRPV3 channels, and this can result in differential desensitization. The desensitization of TRPV3-mediated currents is agonist specific and time dependent [87]. It was reported that most single-thread compounds, such as camphor, borneol and menthol, mainly activate the hot channel TRPV3 at mild temperatures, while at low temperatures, they mainly activate the cold channel TRPM8. This explains why applying peppermint to the skin burns and tingles at high temperatures, and it cools at low temperatures [88,89,90,91]. Cannabinoids and their derivatives (Table 2, entries 13, 14) stimulate the TRPV3-mediated Ca^2+^ influx. Cannabidiol (CBD) and tetrahydrocannabinoids have higher potency and efficacy, with EC_50_ values of 3.8 ± 0.4 µM and 3.7 ± 1.6 µM, respectively [92]. CBD can activate a variety of TRP channels, including TRPV1, TRPV2, TRPV3, TRPV4, TRPM8 and TRPA1. Studies have shown their pharmacological effects on inflammation, pain, cancer, acne, drug dependence, seizure anxiety and Parkinson’s and Alzheimer’s diseases [93,94].

#### 3.1.2. Synthetic Compounds

The first known synthetic small-molecule activator of TRPV3 is 2-Aminoethoxydiphenyl borate (2-APB) (Table 2, entry 15) [95,96]. Hu and colleagues found that the activation mechanism of 2-APB in TRPV3 is distinct from the others. It relied on two essential cytoplasmic residues (His426 and Arg696), which are required for TRPV3 sensitivity to 2-APB, but not to camphor or voltage [95]. Unfortunately, 2-APB is not a specific agonist for TRPV3. In addition to inhibiting IP3-induced Ca^2+^ release and most of the TRP channels, 2-APB can activate TRPV1, TRPV2 and TRPV3 at high concentrations [97,98,99]. Compounds with similar structures to 2-APB, such as diphenylboinic anhydride (DPBA) and drofenine, are also effective TRPV3 agonists (Table 2, entries 16, 17) [100].

#### 3.1.3. Endogenous Substances

Farnesic pyrophosphate (FPP) is a key substance in the isoprene pathway, and it is also the precursor of numerous terpenoids, and it is widely used in combinatorial biology. FPP can be biosynthesized by a series of enzymatic reactions, with acetyl-CoA being used as the initial starting material (Table 2, entry 18). FPP is a specific TRPV3 activator with an EC_50_ value of 0.13 µM [35]. FPP can induce intracellular calcium channel reactions and activate membrane currents in HEK293 cells and primary keratinocytes transfected with human TRPV3. The injection of FPP into mouse paws can induce thermal hypersensitivity reactions and acute injurious behavior. The symptom can be reduced when TRPV3 is knocked down by TRPV3 shRNA [35]. Interestingly, vitamin B3, which is also known as nicotinic acid (NA), can enhance TRPV3 activity while inhibiting TRPV2 and TRPV4 [101]. In addition, NO, a pleiotropic cellular signaling molecule that controls a variety of biological processes, can activate TRPV3 through cysteine nitrosation (Table 2, entry 19) [102]. Inflammatory conditions can further enhance the activity of the TRPV3 channels. The downstream elements of the inflammatory cascade reaction and endogenous ligands such as arachidonic acid and protein kinase C (PKC) can sensitize/activate the TRPV3 channels [103].

### 3.2. TRPV3 Antagonists

#### 3.2.1. Natural Compounds

Recently, Yalan Han et al. identified citrusinine II, an acridone alkaloid isolated from the medicinal plant *Atalantia monophylla* (L.) DC (Table 3, entry 1). It directly interacts with Y564 within the S4 helix of TRPV3 and selectively inhibits the TRPV3 channel with an IC_50_ of 12.43 μM [104]. Studies had shown that subcutaneous injection of citrusinine Ⅱ (143 ng/skin part) almost completely inhibited itching behaviors, and it had an obvious analgesic effect [104]. Coumarin osthole is the main bioactive component of the natural Cnidium monnieri plant, and it is commonly used in traditional Chinese herbal treatments, including anti-itching and anti-dermatitis treatments. Xiaoying Sun et al. showed that osthole was a specific inhibitor of the TRPV3 channel, which can effectively inhibit the itching sensation, with an IC_50_ of 37.0 ± 1.9 μM (Table 3, entry 2) [105]. Recently, Arthur Neuberger et al. further elucidated the binding site and inhibition mechanism of coumarin osthole and TRPV3 by applying cryogenic electron microscopy [106]. Studies have shown that TRPV3 has two binding sites for coumarin osthole in the transmembrane region. One of them is located in a pocket structure formed by the intracellular half of the S1–S4 bundle and the C-terminal portion of the TRP helix, and the other one is located in nexus of the linker domain and pre-S1 region [106]. Coumarin osthole induced the closure of TRPV3 ion channels by binding to these two binding sites and converted the TRPV3 ion channel pore into a previously unknown conformation [106]. Interestingly, these two binding sites coincide with the agonist 2-APB binding site [106]. Hang Qi et al. studied the active components of the herb *Achillea alpine*, isochlorogenic acid A (IAA) and isochlorogenic acid B (IAB) (Table 3, entries 3,4), which can reduce ear swelling, keratinocyte death and chronic itching caused by over-activated TRPV3 [107]. They are selective inhibitors of TRPV3, and the TRPV3 channel opening probabilities decreased from 26.9 ± 5.5% to 3.7 ± 1.2% and 3.2 ± 1.1%, respectively. The IC_50_ values were 2.7 ± 1.3 μM for IAA and 0.9 ± 0.3 μM for IAB. Kewei Wang et al. identified natural forsythoside B as a new selective inhibitor of TRPV3 from *Lamiophlomis rotata*, which can alleviate acute and chronic itching by inhibiting the TRPV3 channel function, with an IC_50_ value of 6.7 ± 0.7 μM (Table 3, entry 5) [108]. Verbascoside, a phenylethanoid, specifically inhibited the TRPV3 channel, with an IC_50_ of 14.1 ± 3.3 μM (Table 3, entry 7) [109]. Marine sponges contain many small-molecule natural products. Among them, guanidine alkaloids have unique structures and diverse biological activities. Of these, the acyclic guanidine alkaloids, pulchranin A, B and C, had inhibitory effects on TRPV3, with the EC_50_ values of 71.8 ± 9.4 μM, 117.9 ± 11.8 μM and >200 μM, respectively (Table 3, entries 8–10) [110,111]. In 2013, Yuliya Korolkova et al. isolated the cycloguanidine alkaloids, monanchomycalin B and urpocidin A, from marine sponges, and they are the antagonists of the TRPV1, TRPV2 and TRPV3 channels. Monanchomycalin B had very good activity, with EC_50_ values of 6.02 µM, 2.84 µM and 3.25 µM for TRPV1, TRPV2 and TRPV3, respectively (Table 3, entry 6) [112].

#### 3.2.2. Synthetic Compounds

Ruthenium red (Table 3, entry 11), a water soluble polycationic dye, is an inhibitor of the TRPV family and TRPA1 and the most common TRPV3 channel inhibitor [113,114]. Interestingly, 2,2-diphenyltetrahydro-furan (DPTHF), an analogue of 2-APB, is also an inhibitor of TRPV3 channels (Table 3, entry 12) [115]. Fang Zhang et al. found the lead compound, 2,6-dimethoxy-n-(4-(trifluoromethyl)phenyl)oxazol-2-ylbenzamide, by performing structure-based virtual screening and further optimized the structure to obtain the more active TRPV3 antagonist N-(5-chloro-4-(4-(trifluoromethyl) pheny l) oxazol-2-yl)-2,6-dimethoxybenzamide (PC5), with an IC_50_ of 2.63 ± 0.28 µM (Table 3, entry 13) [116]. Mengqi Lv et al. analyzed the structure of forsythoside B and proposed that cinnamate ester is the key fragment. A total of 23 targeted compounds were synthesized through a series of chemical modifications. Compound 7C showed the highest TRPV3 inhibitory efficiency, with an IC_50_ value of 1.05 μM, and it showed good selectivity against TRPV1 and TRPV4 channels (Table 3, entry 14) [117].

Local anesthetics play an important role in the treatment of clinical pruritus and the inhibition of various types of pain. To further explore the potential mechanism of the analgesic effect of local anesthetics, it is necessary to investigate the interaction between local anesthetics and TRP ion channels. Recently, Reiko Horishita et al. discovered the interaction between local anesthetics and TRPV3 channels. The results show that the charged form of the local anesthetic interacts with the TRPV3 ion channel outside the cell. The degree of inhibition is positively correlated with the concentration of local anesthetic. The IC_50_ values of bupivacaine, mepivacaine, lidocaine and ropivacaine were 0.69 ± 0.41, 1.4 ± 0.3, 0.28 ± 0.04, 2.5 ± 0.5 and 0.17 ± 0.04 mM, respectively (Table 3, entries 15–18) [118]. Qiang Liu et al. reported that the local anesthetic dyclonine also had TRPV3 inhibitory activity, with an IC_50_ of 3.2 µM (Table 3, entry 19) [119]. A series of studies suggest that the inhibition degree of local anesthetics on TRPV3 ion channels is positively correlated with the concentration. To better understand the molecular mechanism, Arthur Neuberger et al. used cryo-EM to characterize the protein structure of the TRPV3–dyclonine complex [120]. Dyclonine binds to the central void of the ion channels to block the passage of extracellular Ca^2+^, and then, it inhibits the activity of TRPV3 [120]. These findings help us to further explore the mechanisms of local anesthetics, which may be used to treat various diseases caused by TRPV3 agitation.

Hydra Biosciences Inc., Glenmark and Abbvie Inc. have shown intensive interest in the small-molecule inhibitors of TRPV3 channels. Hydra Biosciences reported on a series of tetrahydroquinoline amide TRPV3 inhibitors [121]. Example #64 had an IC_50_ of 0.2–1 μM, and it was highly selective (>40-fold vs. other TRP channels), reducing the heat sensitivity after a carrageenan injection in burned areas or the hind paws (Table 3, entry 20). In addition, the quinazolinone compound, DCP-THQ, showed almost no activity against TRPV1, TRPV4, TRPM8 and TRPA1, with an IC_50_ of 117 nM for TRPV3 (Table 3, entry 21) [122,123]. The Glenmark Pharmaceutical Company described additional quinazolinone analogs, which are similar to the chemical structure previously disclosed by Hydra (Table 3, entry 22) [124]. The Glenmark Pharmaceutical Company further extended the intellectual property rights by describing TRPV3 antagonists of the thiophene [125], chromioylamide [126], chromioketone [127], imidazole [128] and benzimidazole [129] classes (Table 3, entries 23–28). Abbvie reported on the synthesis and biological characteristics of a series of (pyridine-2-yl) methanol derivatives. The dominant molecule, cis-3-[(S)-hydroxy(pyridin-2-yl)methyl]-1-methyl-3-[4-(trifluoromethyl)pyrid-in-2-yl]cyclobutanol (74a), had strong TRPV3 inhibitory activity, with an IC_50_ of 0.38 μM, which was effective in a rat neuropathic pain model (Table 3, entry 29) [130].

#### 3.2.3. Endogenous Substances

Many natural TRPV3 modulators have been reported, but a few endogenous TRPV3 inhibitors have been discovered. A few endogenous lipid metabolites have TRPV3 inhibitory activities. A member of the docosahexaenoic acid-derived lipidolytic family, 17 (*R*)-resolvin D1 (17R-RVD1), was reported to be a specific TRPV3 inhibitor, with an IC_50_ of 0.4 μM, which significantly reduced the level of TRPV3-mediated abnormal pain (Table 3, entry 30) [131]. Interestingly, endogenous TRPV3 agonists, such as the precursor molecule isopentenyl pyrophosphate (IPP), are endogenous TRPV3 antagonists synthesized by the mevalonate pathway (Table 3, entry 31). The IC_50_ values were 0.24 μM and 7.5 μM, respectively, and they performed extensive analgesic actions [132].

**Table 2:** TRPV3 agonists.

Entry	Name	Structure	Activate	Inhibit	EC_50_ for TRPV3	Description/Use	Reference(s)
**Natural compounds**							
1	Incensole acetate (IA)	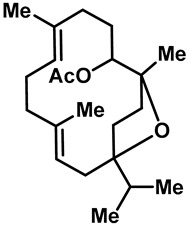	TRPV3	—	16 µM	A *Boswellia* resin component can induce anti-anxiety-like and antidepressant behavior effects in wild-type (WT) mice.	[81]
2	Serratol	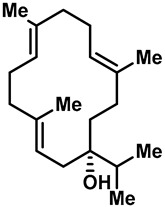	TRPV3	—	0.15 ± 0.01 μM	A TRPV3 agonist, isolated from Indian frankincense, had low retrofitting potential. Its affinity can be significantly improved by the acylation, esterification and oxidation of serratol.	[82]
3	Camphor	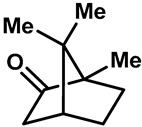	TRPV1 TRPV3 TRPM8	TRPA1	6.03 ± 1.47 mM	Topical analgesia by desensitizing TRPV1; modulated the sensations of warmth in humans.	[78,80,86,131,132]
4	(+)-Borneol	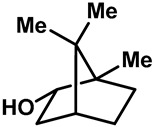	TRPV3 TRPM8	TRPA1	3.45 ± 0.13 mM	Borneol, a common Chinese medicine, had significant analgesic and anti-inflammatory effects by activating TRPM8.	[86,133,134]
5	Menthol	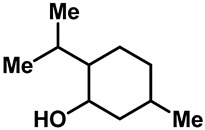	TRPV3 TRPM8 TRPA1 (low)	TRPA1 (high)	1 mM	Menthol mainly activated the hot channel by TRPV3 and the cold channel by TRPM8, and it can induce an analgesic effect by the inhibition of TRPA1.	[135,136]
6	Carvacrol	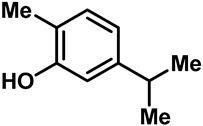	TRPV3 TRPA1	TRPM8	0.49 ± 0.07 mM	Carvacrol is rapidly desensitized by TRPA1.	[63,86,136]
7	Eugenol	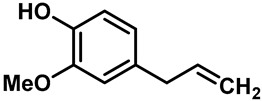	TRPV1 TRPV3 TRPA1	—	2.3 mM	Carvol, isolated from oregano essential oil, is a powerful vasodilator by TRPV3 in the endothelium.	[63,137]
8	Citral	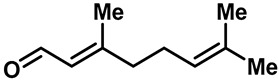	TRPV1 TRPV3 TRPM8 TRPA1	TRPV1-4 TRPM8 TRPA1	926 µM	Citral, a bioactive ingredient in lemongrass, is used to treat abnormal pain, itching or other types of pain involving superficial sensory nerves and skin.	[85]
9	6-*tert*-Butyl-m-cresol	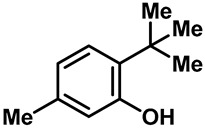	TRPV3	—	0.37 ± 0.1 mM	A much more potent TRPV3 inhibitor than camphor is.	[86]
10	Thymol	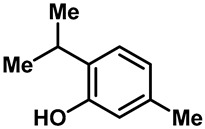	TRPV3 TRPM8 TRPA1	—	0.86 ± 0.07 mM	A TRP channel agonist derived from thyme (*thymus vulgaris*) and oregano (*origanum vulgare*).	[63,86,131]
11	Dihydrocarve-ol	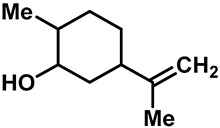	TRPV3	—	2.57 ± 0.42 mM	A potent TRPV3 inhibitor that is used as food additives and artificial spices.	[86]
12	(-)-Carveol	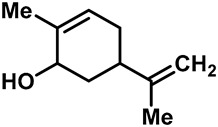	TRPV3	—	3.03 ± 1.16 mM	A potent TRPV3 inhibitor that is used as food additives and artificial spices.	[86]
13	Tetrahydrocannabivarin (THCV)	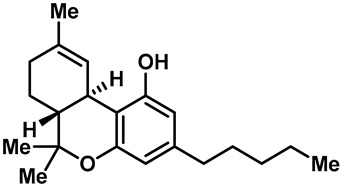	TRPV2 TRPV3 TRPV4	—	3.8 ± 0.4 µM	A non-specific TRPV channel agonist that is used as an anti-tumor, nervous system protection, immunomodulatory, anti-inflammatory and anti-oxidant agent.	[92,138,139]
14	Cannabidiol (CBD)	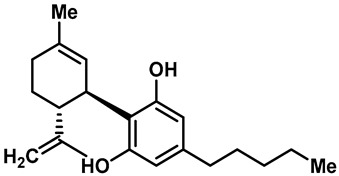	TRPV1 TRPV2 TRPV3 TRPV4 TRPM8 TRPA1	—	3.7 ± 1.6 µM	Cannabidiol has anti-epileptic, immunomodulatory, analgesic, anti-oxidation, anti-convulsive, anti-anxiety and other effects.	[92,140]
**Synthetic compounds**							
15	2-Aminoethoxydiphenyl borate (2-APB)	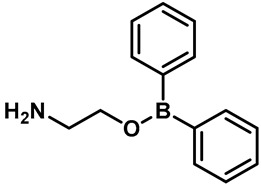	TRPV1 TRPV2 TRPV3	TRPC1-6 TRPM7 TRPM8	28 µM	2-APB can activate TRPV1, TRPV2 and TRPV3 at higher concentrations, and it inhibits the IP3 receptor and TRP channels.	[96]
16	Diphenylborinic anhydride (DPBA)	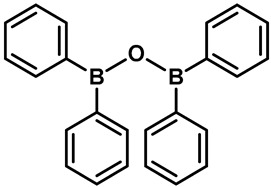	TRPV1 TRPV2 TRPV3 TRPV4	—	85.1 µM	2-APB structural analogue.	[115]
17	Drofenine	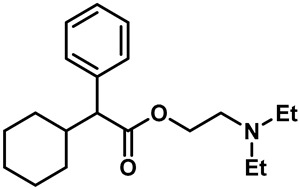	TRPV3	—	207 µM	2-APB structural analogue that can improve TRPV3 selectivity.	[100]
**Endogenous substances**							
18	FPP	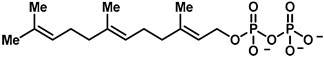		—	0.13 µM	The first endogenous TRPV3 activator.	[35]
19	Nitric oxide	**NO**		—	NA	A pleiotropic cellular signaling molecule that can activate TRPV3 through cysteine nitrosation.	[102]

**Table 3:** TRPV3 antagonists.

Entry	Name	Structure	Activate	Inhibit	IC_50_ for TRPV3	Description/Use	Reference(s)
**Natural compounds**							
1	Citrusinine II	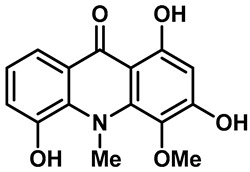	—	TRPV3	12.43 μM	Citrusinine II can selectively inhibit TRPV3 and reduce itchy behavior by interacting with Y564 in the TRPV3 helix.	[104]
2	Osthole	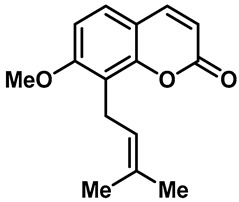	—	TRPV1 TRPV3	37.0 ± 1.9 μM	Cusson, isolated from *Cnidium monnieri* (L.), is used as an antipruritic herbal medicine.	[105,141]
3	Isochlorogenic acid A	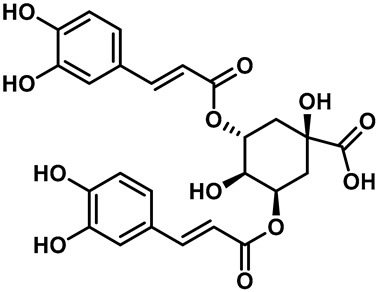	—	TRPV3	2.7 ± 1.3 μM	A TRPV3 specific inhibitor that can significantly reverse ear swelling in dermatitis and chronic pruritus.	[107]
4	Isochlorogenic acid B	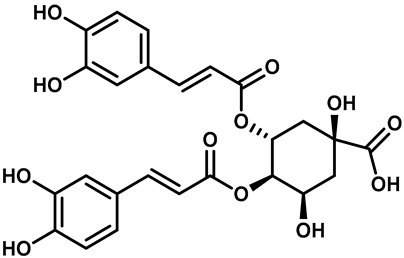	—	TRPV3	0.9 ± 0.3 μM	A TRPV3 specific inhibitor that can significantly reverse ear swelling in dermatitis and chronic pruritus.	[107]
5	Forsythoside B	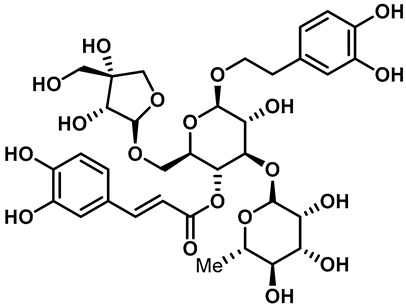	—	TRPV3	6.7 ± 0.7 μM	A TRPV3 inhibitor that can significantly reduce acute pruritus.	[108]
6	Monanchomycalin B	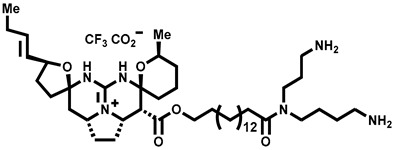	—	TRPV1 TRPV2 TRPV3	3.25 μM	A TRPV inhibitor that is isolated from the marine sponge *Monanchora pulchra*.	[112]
7	Verbascoside	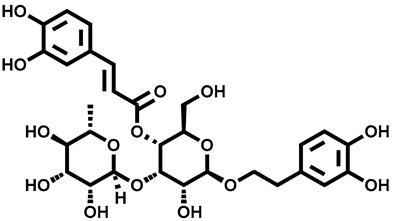	—	TRPV3	14.1 ± 3.3 μM	A TRPV3 inhibitor that can effectively relieve atopic dermatitis when applied topically.	[109]
8	Pulchranin A	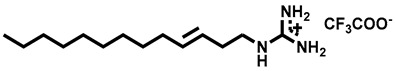	—	TRPV1 TRPV3 TRPA1	71.8 ± 9.4 μM	A moderately potent TRPV1 inhibitor and a minimally potent TRPV3 and TRPA1 inhibitor that is isolated from marine sponge *Monanchora pulchra*.	[110,111]
9	Pulchranin B	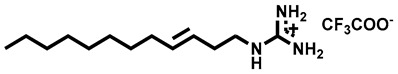	—	TRPV1 TRPV3 TRPA1	117.9 ± 11.8 μM	A moderately potent TRPV1 inhibitor and a minimally potent TRPV3 and TRPA1 inhibitor that is isolated from marine sponge *Monanchora pulchra.*	[110]
10	Pulchranin C	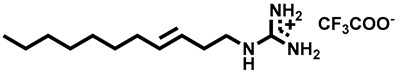	—	TRPV1 TRPV3 TRPA1	>200 μM	A moderate potent TRPV1 inhibitor and a minimally potent TRPV3 and TRPA1 inhibitor that is isolated from marine sponge *Monanchora pulchra*.	[110]
**Synthetic compounds**							
11	Ruthenium Red	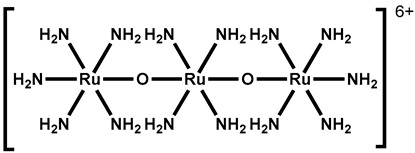	—	TRP(S)	NA	Broad-spectrum, non-selective, cationic channel blockers.	[113]
12	2,2-diphenyltetrahydro-furan (DPTHF)	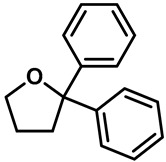	—	TRPV1 TRPV2 TRPV3	6–10 μM	2-APB structural analogue.	[115]
13	PC5	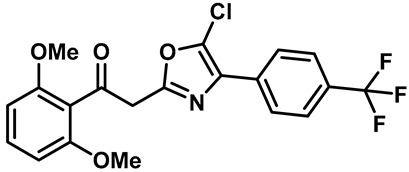	—	TRPV3	2.63 ± 0.28 μM	A TRPV3 inhibitor that is obtained by virtual protein structure screening and lead compound structure optimization.	[116]
14	7C	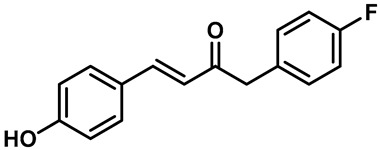	—	TRPV3	1.05 μM	A TRPV3 inhibitor that is obtained by the bioelectron isoarrangement principle.	[117]
15	Bupivacaine	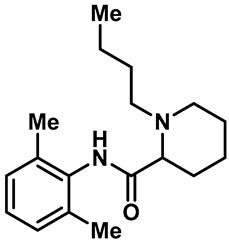	—	TRPV3	0.17 ± 0.04 mM	A TRPV3 inhibitor that can be used as local anesthetics by extracellular interactions of their charged forms with the TRPV3 channel pore.	[118]
16	Mepivacaine	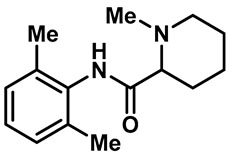	—	TRPV3	1.4 ± 0.3 mM	A TRPV3 inhibitor that can be used as local anesthetics by extracellular interactions of their charged forms with the TRPV3 channel pore.	[118]
17	Lidocaine	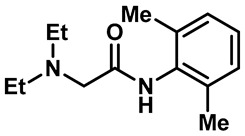	—	TRPV3	2.5 ± 0.5 mM	A TRPV3 inhibitor that can be used as local anesthetics by extracellular interactions of their charged forms with the TRPV3 channel pore.	[118]
18	Ropivacaine	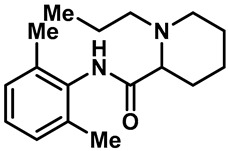	—	TRPV3	0.28 ± 0.04 mM	A TRPV3 inhibitor that can be used as local anesthetics by extracellular interactions of their charged forms with the TRPV3 channel pore.	[118]
19	Dyclonine	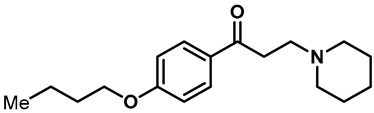	—	TRPV3	3.2 μM	A TRPV3 inhibitor that can ameliorate the hyperactivity caused by itch/scratching behaviors.	[119]
20	Example #64 (WO 2006/122156)	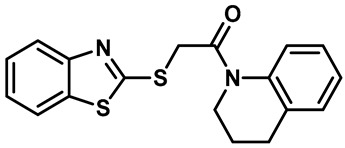	—	TRPV3	0.2–1 μM	A TRPV3 inhibitor discovered by Hydra Biosciences that can reduce heat sensitivity after carrageenan injection in burns or hind paws.	[121]
21	DCP-THQ (WO 2007/056124)	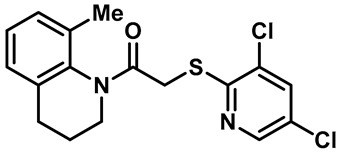	—	TRPV3	117 nM	A TRPV3 inhibitor discovered by Hydra Biosciences.	[123]
22	Example #19 (US 2010/0292554A1)	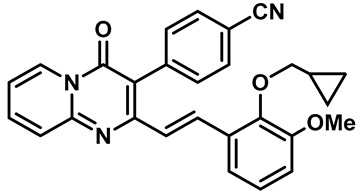	—	TRPV3	200–1000 nM	A TRPV3 inhibitor discovered by Glenmark.	[124]
23	Example #58 (US 2009/0286811A1)	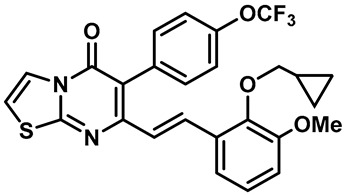	—	TRPV3	< 500 nM	A TRPV3 inhibitor discovered by Glenmark.	[125]
24	Example #58 (WO 2009/130560)	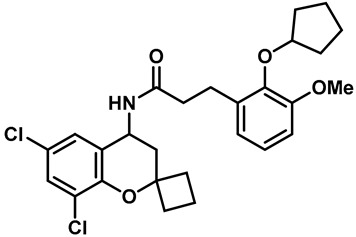	—	TRPV3	<250 nM	A TRPV3 inhibitor discovered by Glenmark.	[126]
25	Example #23 (WO 2010/055384)	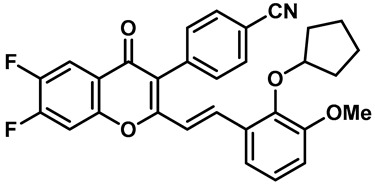	—	TRPV3	<50 nM	A TRPV3 inhibitor discovered by Glenmark.	[127]
26	Example #7 (US2010/0152192)	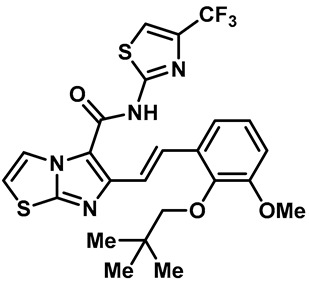	—	TRPV3	<100 nM	A TRPV3 inhibitor discovered by Glenmark.	[128]
27	Example #37 (US2010/0152192)	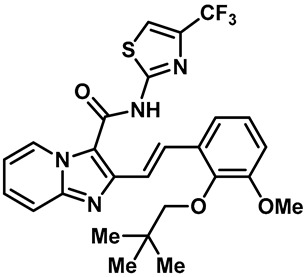	—	TRPV3	<100 nM	A TRPV3 inhibitor discovered by Glenmark.	[128]
28	Example #83 (WO2010/073128)	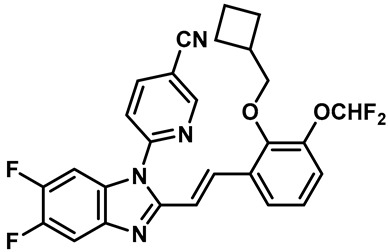	—	TRPV3	<50 nM	A TRPV3 inhibitor discovered by Glenmark.	[129]
29	74a	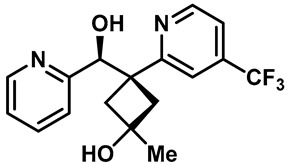	—	TRPV3	0.38 μM	A high potent TRPV3 inhibitor discovered by Glenmark that can be effective in rat neuropathic pain model.	[130]
**Endogenous substance**							
30	17R-RvD1	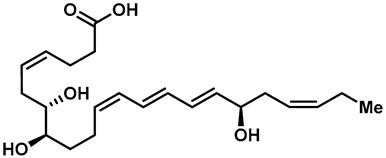	—	TRPV3	0.4 μM	An endogenous TRPV3 inhibitor of endogenous lipid metabolites.	[142]
31	Isopentenyl pyrophosphate (IPP)	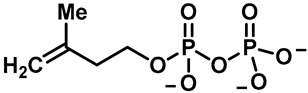	—	TRPV3 TRPA1	0.24 μM	An endogenous TRPA1 and TRPV3 inhibitor for topical analgesia.	[143]

## 4. Conclusions

In summary, the TRPV3 channel is a Ca^2+^-permeable nonselective cation channel. TRPV3 is widely distributed in various organs, including the brain, skin, colon, testis, heart, lung and liver. It has six transmembrane domains, and it forms a tetramer complex. A large number of scientists are studying TRPV3’s role in health and disease. The current studies have revealed that TRPV3 plays a regulatory role in temperature perception, pain transduction, skin physiology, inflammation, cancer and cardiac hypertrophy. TRPV3 channels also play a critical role in skin physiology, including skin barrier formation, hair growth, wound healing, Olmsted syndrome, skin pain and itching. The TRPV3 channel is a potential target for the treatment of itching and skin-related disorders. It is of great significance to search and screen for highly selective TRPV3 activators and inhibitors for beneficial pharmacological interventions for related diseases.

Initially, the activators and inhibitors of TRPV3 were mostly found in extracts of natural products. Early TRPV3 modulators were discovered by accident. Due to the lack of a clear protein structure, the binding site and binding strength of the targeted compounds to TRPV3 were not clear. A recent analysis of the spatial structure of the TRPV3 and ligand complexes provides important guidance for further the rational design and discovery of TRPV3 modulators with high affinity and high selectivity. Through structure-based virtual screening, a series of TRPV3 inhibitors have been designed to accelerate the development of TRPV3 modulators. In addition, TRPV3 has proven to be a highly attractive pharmaceutical target, and several pharmaceutical companies have shown intensive interest in the development of TRPV3 inhibitors.

Though many different approaches have arisen to search for TRPV3 modulators, there are still no commercial drugs of TRPV3 modulators on the market. The reasons for this phenomenon may be: (1) there are some differences in the distribution of drug targets between animals and humans, the mechanism of disease occurrence is not clear, and the mechanism of drug action needs to be improved; (2) the target selection of the drugs is not specific, and it is easy to produce adverse reactions. We need to further improve the method ion channel cloning and screening, refine the molecular theoretical basis of target selectivity and explore the underlying mechanism between specific ion channels and diseases. It lays a foundation for the development of new clinical drugs with TRPV3 regulation and the future study of the physiological function of TRPV3.

## Figures and Tables

**Table 1:** TRPV3 and diseases.

Diseases	Research Evidence	Reference(s)
Olmsted syndrome (OS)	A series of independent clinical reports are identified mutations in the TRPV3 gene as the cause of Olmsted’s syndrome.	[37,38,39,40,41,42]
Pruritic and atopic dermatitis (AD)	1. The expression of TRPV3 is significantly increased in skin lesions and non-diseased skin of patients with specific dermatitis. 2. The pharmacological activation of TRPV3 leads to the development of AD in wild-type mice, and it has no effect on TRPV3 knockout mice. 3. The inhibition of TRPV3 treats inflammatory dorsal skin in a dose-dependent manner.	[43,44,45]
Psoriasis	1. The expression level of TRPV3 in psoriasis patients is significantly higher than it is in people without psoriasis. 2. TRPV3 antagonists relieve the symptoms in patients with moderate-to-severe psoriasis in a dose-dependent manner.	[46]
Cutaneous pruritus	1. TRPV3 knockout mice show no increase in scratching behavior after itch modeling. 2. The expression of TRPV3 is significantly up-regulated in the epidermis of patients with pruritus after having been burnt.	[47,48]
Rosacea	TRPV3 gene expression is significantly increased in rosacea.	[49]
Cancer	1. TRPV3 expression is increased in pancreatic, bone, breast, lung and oral squamous cell cancers. 2. TRPV3 expression is decreased in colorectal cancer.	[21,50,51,52]
Myocardial hypertrophy	1. The expression of TRPV3 is increased in pathological cardiac hypertrophy. 2. TRPV3 expression is increased in cardiomyocyte hypertrophy induced by Ang-II in vitro. 3. TRPV3 inhibitors can significantly aggravate cardiomyocyte hypertrophy and TRPV3 antagonists can slow cardiomyocyte hypertrophy.	[53]
Cardiac fibrosis	TRPV3 activation exacerbate cardiac dysfunction and interstitial fibrosis in pressure-overloaded rats.	[54]
Myocardial infarction	The expression of TRPV3 is significantly up-regulated in neonatal rat cardiomyocytes after myocardial infarction and in hypoxia-treated rats.	[55]
Pain	TRPV3 is overexpressed in skin pain, breast pain, cancer pain and other pain-experiencing tissues.	[56,57,58]
Alopecia	TRPV3 agonists inhibit hair growth, whereas TRPV3 inhibitors significantly reverse the symptom.	[59]

## Data Availability

Not applicable.

## Sample Availability

Samples of the extracts are available from the author.
